# Psychometric Evaluation of the Emotion Regulation Questionnaire (ERQ) in Peruvian Psychology Undergraduates: Factor Structure, Reliability, and Measurement Invariance Across Sex

**DOI:** 10.3390/bs16091711

**Published:** 2026-09-21

**Authors:** Natalia Mavila Guzmán Rodríguez, Jacqueline Roxana Romero Reyna, Paul Alan Arkin Alvarado-García, Patricia Ibeth Tirado Bocanegra, Carlos Miguel Perez Lara

**Affiliations:** 1 Escuela de Psicología, Universidad César Vallejo, Trujillo 13001, Peru; 2 Dirección de Investigación, Universidad Privada de Trujillo, Trujillo 13007, Peru; 3 Escuela de Psicología, Universidad Autónoma del Perú, Lima 15842, Peru; palvaradog@autonoma.edu.pe

**Keywords:** emotion regulation, psychometric properties, measurement invariance, university students, Peru

## Abstract

Evidence for the original adult Emotion Regulation Questionnaire (ERQ) in Peruvian university populations remains limited, particularly regarding score comparability across sex. This cross-sectional psychometric study evaluated the factor structure, internal consistency, and sex-related measurement invariance of the original 10-item ERQ and an exploratory nine-item configuration in 414 psychology undergraduates from a private university in Trujillo, Peru. The correlated two-factor ERQ-10 showed a mixed but viable fit (χ^2^(34) = 84.56, CFI = 0.933, RMSEA = 0.060, 90% CI [0.044, 0.076], SRMR = 0.057), with positive loadings ranging from 0.381 to 0.806. Post hoc exclusion of Item 1 produced a stronger approximate fit (χ^2^(26) = 46.03, CFI = 0.971, RMSEA = 0.043, 90% CI [0.021, 0.063], SRMR = 0.045), but reliability changed negligibly. For the nine-item configuration, full metric invariance was not supported; partial metric, partial scalar, and partial strict invariance were supported after freeing the Item 5 loading across sex. The ERQ-10 therefore remains viable for group-level research in this sample, whereas the alternative configuration should be regarded as a sample-derived hypothesis requiring independent replication, broader samples, and evidence based on external variables.

## 1. Introduction

Emotion regulation refers to the processes through which individuals influence the emotions they experience, when those emotions occur, and how they are expressed or managed. These processes are central to psychological adaptation because they affect subjective well-being, interpersonal functioning, decision-making, and responses to stressful situations (McRae & Gross, 2020). Difficulties in regulating emotions have been consistently associated with symptoms of anxiety, depression, eating disorders, and substance-related problems, supporting the conceptualization of emotion regulation as a transdiagnostic psychological process (Aldao et al., 2010). Nevertheless, the effectiveness of a regulation strategy cannot be understood independently of the context in which it is used, because its psychological consequences may vary according to situational demands, individual characteristics, and cultural norms (M. S. Chen et al., 2025).

Emotion regulation is particularly relevant during university education. Students face increasing academic demands, changes in social relationships, uncertainty about their professional future, and the developmental challenges associated with emerging adulthood. During the transition to university, habitual expressive suppression has been associated with poorer social functioning, less social support, and lower well-being (Srivastava et al., 2009). Consequently, reliable assessment of emotion regulation strategies may contribute to research on university adjustment and mental health and may inform the development and evaluation of preventive and psychoeducational programs.

Accordingly, establishing culturally appropriate and psychometrically defensible measurement of these strategies is an essential foundation for studying psychological processes related to mental health and well-being in university populations.

Within the emotion regulation process model, cognitive reappraisal and expressive suppression are two strategies implemented at different stages of the emotion-generative process. Cognitive reappraisal is an antecedent-focused strategy that involves modifying the interpretation of an emotionally relevant situation before the emotional response is fully developed. Expressive suppression is a response-focused strategy that involves inhibiting the observable expression of an emotion after it has been elicited (Gross & John, 2003). Although cognitive reappraisal is generally associated with more favorable affective and interpersonal outcomes and expressive suppression with less favorable outcomes, these associations should not be interpreted as universally adaptive or maladaptive. Recent cross-cultural evidence indicates that the relationships between both strategies and mental health are moderated by cultural and socioeconomic characteristics (M. S. Chen et al., 2025). 

Gross and John developed the 10-item Emotion Regulation Questionnaire (ERQ) to assess the habitual use of cognitive reappraisal and expressive suppression (Gross & John, 2003). The cognitive reappraisal subscale contains six items, whereas the expressive suppression subscale contains four items, all rated on a seven-point Likert scale. The original study supported a two-factor structure and reported acceptable internal consistency for both subscales. Subsequent research in general community samples has replicated this correlated two-factor structure and demonstrated theoretically consistent associations between ERQ scores, psychological distress, and alexithymia (Preece et al., 2020). 

The ERQ has been translated and examined in several cultural and linguistic contexts. In Argentina, a large-scale adaptation supported the two-factor structure, internal consistency, temporal stability, and associations with anxiety, depression, personality traits, and other emotion regulation processes (delValle et al., 2022). Evidence from Mexico also supported the two-factor model, acceptable internal consistency, and theoretically consistent relationships with empathy and alexithymia (Olalde-Mathieu et al., 2022). Studies conducted among Ecuadorian university students have provided evidence for the factorial structure, reliability, and strict measurement invariance across sex, although some findings have suggested that alternative structural representations may also warrant consideration (Moreta-Herrera et al., 2022). Additional evidence from Argentina has supported the two-factor configuration while identifying variation in the performance of particular items (Vizioli, 2024). Similarly, research among Chinese university students found that an abbreviated eight-item version achieved better fit than the complete version and demonstrated measurement invariance across sex (Zhao et al., 2024). These findings indicate that the ERQ generally retains its conceptual distinction between cognitive reappraisal and expressive suppression but that its item functioning and structural performance may vary across populations.

Cross-cultural evidence also demonstrates that a measurement model that performs adequately in one population cannot be assumed to operate equivalently in another. An evaluation of the ERQ-8 across 29 countries found only configural and metric invariance in a subset of the participating countries, suggesting that the interpretation and functioning of emotion regulation items may be influenced by cultural context (Burghart et al., 2023). Consequently, context-specific psychometric evaluation remains necessary. Furthermore, measurement invariance must be established before observed scores or latent means are meaningfully compared between groups, because apparent group differences may otherwise reflect nonequivalent item functioning rather than differences in the underlying construct (Putnick & Bornstein, 2016). 

The evaluation of an instrument should therefore extend beyond global model fit. Evidence based on internal structure should be complemented by appropriate estimates of score reliability and analyses of measurement equivalence across relevant groups. Cronbach’s alpha remains widely reported, but it relies on assumptions that are often difficult to satisfy in multidimensional or congeneric measurement models. McDonald’s omega provides a complementary estimate based more directly on the factor loadings and error variances of the measurement model (Dunn et al., 2014). Reporting both coefficients offers a more informative assessment of the internal consistency of cognitive reappraisal and expressive suppression scores.

In Peru, the ERQ was previously linguistically adapted and evaluated in 320 university students from two private universities. That study supported the original two-factor structure and reported Cronbach’s alpha coefficients of 0.72 for cognitive reappraisal and 0.74 for expressive suppression (Gargurevich & Matos, 2010). More recently, a simplified nine-item questionnaire was evaluated in 312 Peruvian psychology students (Valencia et al., 2026). Although it retained cognitive reappraisal and expressive suppression, the instrument differed from the original adult ERQ in its item composition and response format and did not examine multigroup measurement invariance across sex. Consequently, neither previous Peruvian study established whether scores from the original adult ERQ or from a data-derived alternative configuration were sufficiently comparable between women and men.

Accordingly, this study evaluated whether the original 10-item adult ERQ reproduced its expected correlated two-factor structure and yielded sufficiently reliable subscale scores in Peruvian psychology undergraduates. It also examined the extent to which the measurement model supported by the preceding analyses was invariant across sex. Any data-driven alternative configuration was treated as exploratory rather than as a prespecified replacement for the ERQ-10. A secondary, conditional objective was to summarize sample-specific score distributions for the configuration supported by the analyses.

## 2. Materials and Methods

### 2.1. Study Design and Setting

A cross-sectional psychometric study was conducted to examine evidence based on the internal structure of the Emotion Regulation Questionnaire (ERQ), the internal consistency of its subscale scores, and measurement invariance across sex. The study was conducted among undergraduate psychology students enrolled at a private university in Trujillo, a city in northern Peru. Recruitment was limited to psychology undergraduates because this population was accessible to the research team and permitted standardized, supervised classroom administration under comparable conditions. This feasibility-based sampling decision was not intended to produce a representative sample of the wider Peruvian university population.

### 2.2. Participants

The final analytical sample comprised 414 undergraduate psychology students recruited through non-probability convenience sampling. Of these, 219 were women (52.9%) and 195 were men (47.1%), and their mean age was 19.78 years (SD = 2.36). Two-thirds of the participants were enrolled in the first four academic semesters (66.7%).

Students were eligible to participate if they were enrolled in the undergraduate psychology program at the time of data collection, voluntarily agreed to participate, provided written informed consent, and returned the study protocol. Before analysis, all protocols were screened for missing or illegible responses. The final analytical database contained one missing response to ERQ Item 3, representing 0.02% of the 4140 possible ERQ item responses; all other ERQ responses were complete.

### 2.3. Instrument

Emotion regulation was assessed using the Spanish-language version of the Emotion Regulation Questionnaire (ERQ), originally developed by Gross and John (2003) and adapted for the Peruvian context by Gargurevich and Matos (2010). The ERQ is a 10-item self-report questionnaire designed to assess two conceptually distinct emotion-regulation strategies: cognitive reappraisal and expressive suppression.

The Cognitive Reappraisal subscale comprises six items (Items 1, 3, 5, 7, 8, and 10) and evaluates the extent to which individuals modify their interpretation of an emotional situation to change its emotional impact. The Expressive Suppression subscale consists of four items (Items 2, 4, 6, and 9) and measures the inhibition of the outward expression of emotions after an emotional response has already been generated.

Items are rated on a seven-point Likert-type scale ranging from 1 (“strongly disagree”) to 7 (“strongly agree”). In the original 10-item version, cognitive reappraisal scores are calculated by summing Items 1, 3, 5, 7, 8, and 10 and range from 6 to 42. Expressive suppression scores are calculated by summing Items 2, 4, 6, and 9 and range from 4 to 28. Higher scores indicate more frequent use of the corresponding emotion-regulation strategy. Because cognitive reappraisal and expressive suppression represent conceptually distinct strategies, a total ERQ score was not calculated.

For the exploratory nine-item configuration examined in subsequent analyses, the cognitive reappraisal score comprised Items 3, 5, 7, 8, and 10 and ranged from 5 to 35. The expressive suppression score retained Items 2, 4, 6, and 9 and continued to range from 4 to 28. This exact item composition is reported because it differs from previously published ERQ-9 configurations.

A brief sociodemographic form recorded age, sex, academic semester, and district of residence. No measure of mental health, emotional symptoms, psychiatric diagnosis, or treatment status was administered.

### 2.4. Procedure

Data were collected between September and October 2025 after authorization from the university and approval from the corresponding research ethics committee. Potential participants were approached during regularly scheduled classroom sessions. Before administering the questionnaires, the researchers explained the study objectives, the voluntary nature of participation, and the confidential treatment of the information collected.

Students who agreed to participate signed a written informed consent form and subsequently completed the ERQ and the sociodemographic questionnaire collectively and in person inside their classroom. Administration conditions and instructions were kept consistent across groups. No financial or non-financial incentive or compensation was provided. Participants could decline or withdraw without giving a reason. Completed questionnaires were coded to protect identity, and no directly identifying information was incorporated into the analytical database.

### 2.5. Statistical Analysis

Data were entered, coded, and checked for inconsistencies before analysis. Descriptive statistics were calculated for each ERQ item, including the mean, standard deviation, skewness, and kurtosis.

Confirmatory factor analysis (CFA) was conducted to evaluate the internal structure of the ERQ. For the original ERQ-10 and the exploratory nine-item configuration excluding Item 1, three theoretically relevant models were evaluated: (a) a unidimensional model; (b) an orthogonal two-factor model in which cognitive reappraisal and expressive suppression were uncorrelated; and (c) a correlated two-factor model. Each item loaded only on its designated factor and residuals were not allowed to correlate. Nested comparisons were made only between models containing the same observed indicators. Fit indices for ERQ-10 and the nine-item configuration were compared descriptively because the models contained different item sets. Examination of Item 1 considered its standardized loading, corrected item-total correlation, changes in alpha and omega, content coverage, and the post hoc nature of any deletion; no single numerical cutoff was treated as sufficient evidence for removal.

The CFA and multigroup measurement-invariance models were estimated using IBM SPSS Amos, version 30.0 (IBM Corp., Armonk, NY, USA), with maximum likelihood estimation. The single missing response to Item 3 was handled using full-information maximum likelihood; no item-level imputation was performed, and all 414 participants were retained. Maximum likelihood was retained because every ERQ item used seven ordered response categories and the observed distributions showed no severe univariate non-normality; absolute skewness and kurtosis were below 1.00 for all items. Simulation evidence indicates that continuous and categorical estimators often perform similarly with six or seven categories under non-extreme distributional conditions, although ordinal estimators may offer advantages for some parameters (Flora & Curran, 2004; Rhemtulla et al., 2012). Estimator choice was therefore considered explicitly when interpreting the findings.

Model fit was evaluated using the chi-square statistic (χ^2^), comparative fit index (CFI), root mean square error of approximation (RMSEA) with its 90% confidence interval, and standardized root mean square residual (SRMR). In accordance with commonly used recommendations, CFI values close to or greater than 0.95, RMSEA values close to or below 0.06, and SRMR values close to or below 0.08 were regarded as evidence of favorable approximate fit (Hu & Bentler, 1999). These values were treated as interpretative guidelines rather than rigid decision rules. Model interpretation also considered standardized loadings, theoretical consistency, parsimony, reliability, content coverage, and whether a model modification had been specified a priori or derived from the same sample.

The internal consistency of cognitive reappraisal and expressive suppression scores was evaluated separately using Cronbach’s alpha (α) and McDonald’s omega (ω). Coefficients were calculated for the original ERQ-10 subscales and, for cognitive reappraisal, for the exploratory configuration excluding Item 1. Omega was derived from standardized factor loadings and residual variances. Reporting both coefficients provides a more informative assessment because alpha assumes essentially equivalent item loadings, whereas omega accommodates differences in the strength of item-factor relations (Dunn et al., 2014). Coefficients near 0.70 were used as general reference values, while considering the small number of items in each subscale.

Measurement invariance across sex was examined for the exploratory nine-item configuration through multigroup CFA. Configural, metric, scalar, and strict models were tested sequentially. Invariance was evaluated primarily through changes in approximate fit because chi-square difference tests are sensitive to sample size. A decrease in CFI not exceeding 0.010 and an increase in RMSEA not exceeding 0.015 supported invariance between successive models (F. Chen, 2007). When full metric invariance was not supported, sex-specific configural estimates were inspected and the loading with the largest absolute between-group discrepancy was freely estimated. Partial scalar and partial strict invariance were then examined while retaining that loading unconstrained. As secondary descriptive analyses, empirical percentiles were calculated for the exploratory nine-item scores, and four score quadrants were formed by cross-classifying cognitive reappraisal and expressive suppression as at or above versus below their respective sample medians. These quadrants were treated only as sample-relative descriptions, not as clinical categories, normative levels, or diagnostic cutoffs. Statistical tests used a two-sided significance level of *p* < 0.05 where applicable.

### 2.6. Ethical Considerations

The study was conducted in accordance with the ethical principles of the Declaration of Helsinki. The research protocol was reviewed and approved by the Research Ethics Committee of the School of Psychology at Universidad César Vallejo (Approval No. 01218-2024/CEI-PSI, dated 11 December 2024). All participants received information about the purpose and procedures of the study and provided written informed consent before completing the questionnaires. Participation was voluntary, and the confidentiality and anonymity of the collected information were protected throughout data collection, analysis, and reporting.

## 3. Results

### 3.1. Participant Characteristics

The analytical sample included 414 psychology undergraduates (219 women, 52.9%; 195 men, 47.1%), with a mean age of 19.78 years (SD = 2.36). Two-thirds of the participants were enrolled in the first four academic semesters (66.7%). One response to Item 3 was missing (0.02% of the 4140 possible ERQ item responses); all other item responses were complete. Full-information maximum likelihood was used for the confirmatory factor and measurement-invariance analyses. Participant characteristics are summarized in Table 1, and participants’ districts of residence are reported in Table S1.

### 3.2. Item Descriptive Statistics and Factor Loadings

Across all 10 items, means ranged from 2.92 to 4.48 and standard deviations from 1.31 to 1.60; absolute skewness and kurtosis values were below 1.00. In the correlated ERQ-10 model, standardized loadings ranged from 0.381 to 0.806. Item 1 had the lowest loading (λ = 0.381) and the lowest corrected item-total correlation within cognitive reappraisal (r = 0.321). Excluding Item 1 was therefore examined as a post hoc sensitivity configuration rather than as an automatic deletion based on a 0.40 cutoff.

In the correlated nine-item model, loadings ranged from 0.474 to 0.808. The complete ERQ-10 loadings and corresponding nine-item estimates are presented together in Table 2. Removing Item 1 slightly increased alpha for cognitive reappraisal from 0.717 to 0.721 but changed omega from 0.728 to 0.727, indicating no material reliability gain.

### 3.3. Confirmatory Factor Analysis and Model Comparison

The unidimensional models performed poorly for both item sets. For the ERQ-10, allowing the two factors to correlate improved fit relative to the orthogonal model, Δχ^2^(1) = 18.35, *p* < 0.001. The correlated ERQ-10 model showed mixed but viable approximate fit: RMSEA and SRMR met commonly used guidelines, whereas CFI remained below the preferred 0.95 value. The same pattern of comparative improvement was observed for the nine-item configuration, for which the correlated model fit better than the orthogonal model, Δχ^2^(1) = 15.40, *p* < 0.001, and showed favorable approximate fit (Table 3). The factor correlations were positive and modest (r = 0.276 for ERQ-10; r = 0.254 for ERQ-9). The interpretation of cognitive reappraisal and expressive suppression as separate subscales was based on their theoretical specification and the comparative model results, not on correlation magnitude alone. The nine-item correlated model had a descriptively stronger fit than the ERQ-10 model; however, no formal comparison was made across item sets because they contained different observed indicators.

### 3.4. Internal Consistency

Internal consistency estimates for the ERQ subscales are presented in Table 4. For cognitive reappraisal, Cronbach’s α was 0.717 and McDonald’s ω was 0.728 for the ERQ-10, whereas α was 0.721 and ω was 0.727 for the exploratory nine-item configuration. For expressive suppression, α was 0.703 and ω was 0.710.

### 3.5. Measurement Invariance Across Sex

The configural model showed an acceptable fit. Constraining all factor loadings to equality produced ΔCFI = −0.0116, which slightly exceeded the prespecified absolute-change criterion of 0.010, although ΔRMSEA remained small. Full metric invariance was therefore not supported. In the sex-specific configural models, Item 5 showed the largest absolute loading discrepancy (men λ = 0.617; women λ = 0.345; |Δλ| = 0.272). After this loading was freed, the partial metric model differed negligibly from the configural model (ΔCFI = −0.0006; ΔRMSEA = −0.0020). The subsequent partial scalar and partial strict models also met the change criteria (Table 5). Accordingly, the nine-item configuration supported partial metric invariance, partial scalar invariance, and partial strict invariance across sex, with the Item 5 loading freely estimated.

### 3.6. Sample-Specific Score Distributions

Empirical percentile values were calculated to describe the exploratory nine-item subscale scores in this sample. The median was 15 for both cognitive reappraisal and expressive suppression, and the 5th–95th percentile ranges were 8–23 and 7–22, respectively (Table 6). These values are not population norms, diagnostic thresholds, or clinical cutoff scores.

Among 413 participants with complete scores on both subscales, 135 (32.7%) scored at or above the sample median on both dimensions, 86 (20.8%) did so only for cognitive reappraisal, 90 (21.8%) did so only for expressive suppression, and 102 (24.7%) scored below both medians. These four quadrants are sample-relative descriptions rather than validated high/low categories. Mental-health profiles could not be compared across the quadrants because no mental-health measure was administered.

## 4. Discussion

This study evaluated the original adult ERQ-10 and an exploratory nine-item configuration in Peruvian psychology undergraduates. The correlated ERQ-10 model yielded mixed but viable evidence: all loadings were positive, RMSEA and SRMR met commonly used guidelines, and CFI was below the preferred 0.95 value. Excluding Item 1 produced a descriptively stronger approximate fit, but internal consistency changed negligibly and the modification reduced content coverage. The ERQ-10 should therefore not be dismissed, while the nine-item configuration should be interpreted as a sample-derived sensitivity result rather than as a definitive replacement.

The comparative model results supported the expected distinction between antecedent-focused cognitive reappraisal and response-focused expressive suppression (Gross & John, 2003). For both item sets, unidimensional models performed poorly and correlated two-factor models fit significantly better than their orthogonal counterparts. The factor correlations were modest (r = 0.276 for ERQ-10 and r = 0.254 for ERQ-9), but their magnitude alone does not establish that the strategies are distinguishable. The interpretation of separate subscales rests jointly on the process-model distinction, the prespecified item assignment, and the comparative fit pattern. These findings are broadly consistent with two-factor evidence from Australian community and Spanish-speaking samples (delValle et al., 2022; Olalde-Mathieu et al., 2022; Preece et al., 2020).

The behavior of Item 1 requires a multiple-criterion interpretation. Its ERQ-10 loading (0.381) and corrected item-total correlation within cognitive reappraisal (0.321) were the lowest for that subscale, and excluding it improved approximate fit. Nevertheless, a loading of 0.381 is not inherently unacceptable. Moreover, alpha increased only from 0.717 to 0.721 and omega changed from 0.728 to 0.727, while removal reduced representation of positive-emotion up-regulation. The item was therefore examined as a post hoc sensitivity decision, not declared invalid. Published alternatives are also heterogeneous: some ERQ-9 studies removed Item 3 (Rice et al., 2018; Spaapen et al., 2014), whereas Zhao et al. (2024) reported an ERQ-8 excluding Items 1 and 3. This variability argues against treating any single deletion as universally preferable.

Several explanations are possible. Items 1 and 3 both describe changing the content of thought but differ in whether the goal is to increase positive emotion or reduce negative emotion. Their functioning may consequently depend on respondents’ interpretation of emotional valence, the semantic nuances of the translated wording, or the perceived distinction between modifying a thought and reinterpreting an emotional situation. Removing Item 1 may reduce redundancy in the present sample, but it also changes the content balance of the cognitive reappraisal subscale by reducing the representation of positive-emotion up-regulation. Cultural differences in emotion-related beliefs and display norms may also influence how these statements are understood, although the present design cannot directly test this explanation (M. S. Chen et al., 2025; Van Doren et al., 2021). Cognitive interviews and differential item functioning analyses are therefore needed to determine whether the weak loading reflects linguistic interpretation, cultural relevance, redundancy, or sample-specific variation.

Consequently, the stronger approximate fit after removing Item 1 does not demonstrate that the nine-item configuration is generally superior to the ERQ-10. The modification was data-driven and evaluated in the same sample in which the weak item was identified, creating a risk of capitalization on sample-specific characteristics (MacCallum et al., 1992). The ERQ-10 remains the established and more content-complete instrument. The alternative configuration should be identified by its exact composition—cognitive reappraisal Items 3, 5, 7, 8, and 10 and expressive suppression Items 2, 4, 6, and 9—and treated as a hypothesis for independent replication.

The internal-consistency findings reinforce this balanced interpretation. Cognitive reappraisal reliability was nearly identical for ERQ-10 (α = 0.717; ω = 0.728) and the nine-item configuration (α = 0.721; ω = 0.727), while expressive suppression yielded α = 0.703 and ω = 0.710. Thus, item removal improved approximate fit without a material reliability gain. These coefficients may be adequate for preliminary group-level research, but they do not support high-stakes individual assessment or clinical decisions. Internal consistency also does not establish temporal stability, conditional precision, or sensitivity to change (Dunn et al., 2014; McNeish, 2018). 

The measurement-invariance results require similarly qualified interpretation. Configural invariance indicated that the same general two-factor pattern was identifiable among women and men. Full metric invariance narrowly failed because ΔCFI was −0.0116, whereas ΔRMSEA remained small. Item 5 had the largest loading discrepancy between men and women, and freeing this parameter supported partial metric invariance. The subsequent models supported partial scalar invariance and partial strict invariance while the Item 5 loading remained unconstrained. Thus, the findings do not establish full measurement equivalence, but they suggest that most parameters operated comparably across sex in this sample.

Item 5 refers to cognitively reframing a stressful situation to remain calm. Its non-invariant loading does not necessarily imply that the item should be eliminated; instead, it indicates that the item contributes differently to the cognitive reappraisal factor across the two groups. Partial measurement invariance can permit appropriately specified comparisons when the non-invariant parameter is explicitly modeled, but it represents weaker evidence than full invariance (Byrne et al., 1989; Putnick & Bornstein, 2016). Therefore, the results should not be described as demonstrating complete measurement equivalence or unrestricted fairness across sex. Any subsequent latent mean comparison should retain the Item 5 loading as freely estimated, and raw-score differences should be interpreted cautiously. It is also important that the present study did not test whether women and men differed in their latent or observed mean levels; it tested only whether the measurement model operated sufficiently similarly across groups.

The partial-invariance result differs from studies that have reported stronger sex or gender invariance for ERQ scores in Ecuadorian university students, Taiwanese college students, Chinese university students, and a United States community sample (Li & Wu, 2020; Moreta-Herrera et al., 2022; Preece et al., 2021; Zhao et al., 2024). Nevertheless, it is consistent with broader evidence that ERQ measurement properties are not completely uniform across populations. For example, an evaluation of the ERQ-8 across 29 countries found acceptable configural and metric invariance in only 14 countries and did not establish scalar invariance across those countries (Burghart et al., 2023). Cross-cultural work comparing the United States and India has likewise demonstrated the importance of evaluating the equivalence of emotion-regulation measures rather than assuming that apparently similar scores have identical meanings across cultural groups (Van Doren et al., 2021). The present findings therefore add to evidence that measurement invariance is an empirical property of scores in a particular population and version of an instrument, rather than a permanent characteristic of the ERQ.

The percentile values and median-based quadrants provide transparent descriptions of score distributions in the study sample. Approximately one third of complete cases scored at or above both sample medians, while the other participants were distributed across the remaining three quadrants. These categories do not represent validated high or low emotion regulation, population norms, or clinical thresholds. Because mental or emotional health was not measured, the study also cannot determine whether the quadrants differed in psychological distress or well-being.

These strengths must be considered alongside important limitations. First, recruitment was restricted to psychology students because this population was accessible and allowed consistent classroom administration, but the single-university convenience sample is not representative of all Peruvian university students. Generalization to other disciplines, public universities, regions, non-student adults, or clinical populations is therefore limited. Second, Item 1 was removed post hoc and the resulting configuration was not cross-validated independently. Third, no measure of mental health, well-being, alexithymia, personality, or another emotion-regulation construct was administered, so relationships with external variables and the clinical meaning of score profiles could not be examined. Fourth, test–retest reliability, predictive validity, and sensitivity to change were not evaluated. Fifth, the self-report design may be affected by response styles and social desirability. Finally, although seven-category items can often be analyzed as approximately continuous, replication with ordinal estimators based on polychoric correlations remains advisable (Flora & Curran, 2004; Rhemtulla et al., 2012). 

Future studies should preregister and test the original ERQ-10 and alternative configurations in independent, multi-university samples that include students from different disciplines and regions. Direct comparison of the ERQ-10, the present configuration excluding Item 1, the published ERQ-9 excluding Item 3, and the ERQ-8 excluding both items could clarify whether item performance is stable or sample-specific. Ordinal CFA, item response theory, differential item functioning, and cognitive interviewing could further examine Items 1, 3, and 5. Longitudinal designs and associations with theoretically relevant external variables are also needed to establish temporal stability and convergent, discriminant, criterion, and predictive validity.

## 5. Conclusions

This study evaluated the factor structure, internal consistency, and sex-related measurement equivalence of the ERQ in Peruvian psychology undergraduates. The correlated ERQ-10 showed mixed but viable fit, whereas a post hoc configuration excluding Item 1 showed a stronger approximate fit without a material reliability gain. For the nine-item configuration, partial metric invariance, partial scalar invariance, and partial strict invariance were supported after the Item 5 loading was freely estimated across sex. The ERQ-10 therefore remains suitable for cautious group-level research in this sample and should not be replaced on the basis of these data alone. The alternative configuration is hypothesis-generating and requires independent replication. Neither configuration is supported here for individual clinical decisions, and broader samples, ordinal analyses, temporal stability, and evidence based on external variables are needed before wider use can be recommended.

## Figures and Tables

**Table 1:** Participant characteristics (N = 414).

Characteristic	Category	M (SD) or *n* (%)
Age, years	—	19.78 (2.36)
Sex	Women	219 (52.9)
	Men	195 (47.1)
Academic semester	1–2	127 (30.7)
	3–4	149 (36.0)
	5–6	46 (11.1)
	7–8	91 (22.0)
	9–10	1 (0.2)

Note. Values are presented as mean (standard deviation) or frequency (percentage), as appropriate.

**Table 2:** Item descriptive statistics and standardized factor loadings.

Subscale	Item	*n*	M	SD	Skewness	Kurtosis	λ ERQ-10	λ ERQ-9
CR	1	414	3.26	1.53	0.59	0.20	0.381	—
ES	2	414	3.26	1.57	0.26	−0.59	0.571	0.569
CR	3	413	2.92	1.45	0.61	0.18	0.498	0.474
ES	4	414	4.48	1.55	−0.26	−0.27	0.580	0.579
CR	5	414	3.00	1.36	0.56	0.39	0.465	0.484
ES	6	414	3.86	1.60	0.14	−0.41	0.806	0.808
CR	7	414	3.05	1.31	0.60	0.44	0.679	0.688
CR	8	414	3.00	1.35	0.63	0.56	0.678	0.685
ES	9	414	3.43	1.56	0.28	−0.32	0.493	0.492
CR	10	414	2.94	1.36	0.70	0.70	0.611	0.604

Note. CR = cognitive reappraisal; ES = expressive suppression; M = mean; SD = standard deviation; λ = standardized loading in the correlated two-factor model. A dash indicates that Item 1 was not included in the exploratory nine-item configuration.

**Table 3:** Fit indices for ERQ-10 and exploratory nine-item measurement models.

Item Set and Model	χ^2^ (df)	CFI	RMSEA [90% CI]	SRMR
ERQ-10 unidimensional	329.77 (35)	0.610	0.143 [0.129, 0.157]	0.127
ERQ-10 orthogonal two-factor	102.91 (35)	0.910	0.069 [0.053, 0.084]	0.093
ERQ-10 correlated two-factor	84.56 (34)	0.933	0.060 [0.044, 0.076]	0.057
ERQ-9 unidimensional	297.32 (27)	0.602	0.156 [0.140, 0.172]	0.139
ERQ-9 orthogonal two-factor	61.43 (27)	0.949	0.056 [0.037, 0.074]	0.084
ERQ-9 correlated two-factor	46.03 (26)	0.971	0.043 [0.021, 0.063]	0.045

Note. CFI = comparative fit index; RMSEA = root mean square error of approximation; CI = confidence interval; SRMR = standardized root mean square residual. Formal nested comparisons were conducted only within the same item set.

**Table 4:** Internal consistency of the ERQ subscale scores.

Subscale and Configuration	Items	Cronbach’s α	McDonald’s ω
Cognitive reappraisal, ERQ-10	6	0.717	0.728
Cognitive reappraisal, ERQ-9	5	0.721	0.727
Expressive suppression	4	0.703	0.710

Note. α = Cronbach’s alpha; ω = McDonald’s omega. Alpha estimates involving Item 3 were computed from 413 complete subscale records; omega was estimated with full-information maximum likelihood using all 414 participants.

**Table 5:** Measurement invariance of the ERQ-9 across sex.

Model	Constraints	χ^2^ (df)	CFI	RMSEA [90% CI]	ΔCFI	ΔRMSEA	Decision
M1 Configural	Same pattern	90.38 (52)	0.945	0.042 [0.027, 0.057]	—	—	Supported
M2 Full metric	All loadings equal	105.45 (59)	0.934	0.044 [0.030, 0.057]	−0.0116	+0.0014	Not supported
M2a Partial metric	Item 5 loading free	96.82 (58)	0.944	0.040 [0.026, 0.054]	−0.0006	−0.0020	Supported
M3 Partial scalar	Intercepts equal; Item 5 loading free	104.74 (65)	0.943	0.039 [0.024, 0.052]	−0.0013	−0.0018	Supported
M4 Partial strict	Residuals equal; Item 5 loading free	118.92 (74)	0.936	0.038 [0.025, 0.051]	−0.0074	−0.0001	Supported

Note. CI = confidence interval. Changes for M2 are relative to M1; M2a to M1; M3 to M2a; and M4 to M3. Invariance was supported when ΔCFI ≥ −0.010 and ΔRMSEA ≤ 0.015. Item 5 was retained but its loading was freely estimated across sex.

**Table 6:** Sample-specific empirical percentile values for the exploratory nine-item subscale scores.

Percentile	Cognitive Reappraisal	Expressive Suppression
5	8	7
10	9	9
15	10	11
20	11	11
25	12	12
30	13	13
35	13	13
40	14	14
45	14	14
50	15	15
55	15	16
60	16	16
65	16	17
70	17	18
75	17	18
80	18	19
85	19	20
90	21	21
95	23	22

Note. Cognitive reappraisal percentiles were calculated from 413 complete scores because one participant had a missing response to Item 3. Expressive suppression percentiles were calculated from 414 scores. These values are descriptive and specific to the present sample; they should not be considered population norms or clinical cutoff scores.

## Data Availability

The de-identified data supporting the findings of this study are available from the corresponding author upon reasonable request. The data are not publicly available to protect participant confidentiality and to comply with applicable institutional ethical requirements.

## Supplementary Materials

The following supporting information can be downloaded at: https://www.mdpi.com/article/10.3390/bs16091711/s1, Table S1: Participants’ district of residence.

## References

[B1-behavsci-16-01711] Aldao A., Nolen-Hoeksema S., Schweizer S. (2010). Emotion-regulation strategies across psychopathology: A meta-analytic review. Clinical Psychology Review.

[B2-behavsci-16-01711] Burghart M., Sahm A. H. J., Mier D. (2023). Investigating measurement invariance of the Emotion Regulation Questionnaire-8 (ERQ-8) across 29 countries. Current Psychology.

[B3-behavsci-16-01711] Byrne B. M., Shavelson R. J., Muthén B. (1989). Testing for the equivalence of factor covariance and mean structures: The issue of partial measurement invariance. Psychological Bulletin.

[B4-behavsci-16-01711] Chen F. (2007). Sensitivity of Goodness of fit indexes to lack of measurement invariance. Structural Equation Modeling: A Multidisciplinary Journal.

[B5-behavsci-16-01711] Chen M. S., Cai Q., Omari D., Sanghvi D. E., Lyu S., Bonanno G. A. (2025). Emotion regulation and mental health across cultures: A systematic review and meta-analysis. Nature Human Behaviour.

[B6-behavsci-16-01711] delValle M. V., Andrés M. L., Urquijo S., Zamora E. V., Mehta A., Gross J. J. (2022). Argentinean adaptation and psychometric properties of the Emotion Regulation Questionnaire (ERQ). Psychological Reports.

[B7-behavsci-16-01711] Dunn T. J., Baguley T., Brunsden V. (2014). From alpha to omega: A practical solution to the pervasive problem of internal consistency estimation. British Journal of Psychology.

[B8-behavsci-16-01711] Flora D. B., Curran P. J. (2004). An empirical evaluation of alternative methods of estimation for confirmatory factor analysis with ordinal data. Psychological Methods.

[B9-behavsci-16-01711] Gargurevich R., Matos L. (2010). Propiedades psicométricas del cuestionario de autorregulación emocional adaptado para el Perú. Revista de Psicología (Trujillo).

[B10-behavsci-16-01711] Gross J. J., John O. P. (2003). Individual differences in two emotion regulation processes: Implications for affect, relationships, and well-being. Journal of Personality and Social Psychology.

[B11-behavsci-16-01711] Hu L., Bentler P. M. (1999). Cutoff criteria for fit indexes in covariance structure analysis: Conventional criteria versus new alternatives. Structural Equation Modeling: A Multidisciplinary Journal.

[B12-behavsci-16-01711] Li C.-H., Wu J.-J. (2020). Psychometric evaluation of the Chinese version of the emotion regulation questionnaire in taiwanese college students. Assessment.

[B13-behavsci-16-01711] MacCallum R. C., Roznowski M., Necowitz L. B. (1992). Model modifications in covariance structure analysis: The problem of capitalization on chance. Psychological Bulletin.

[B14-behavsci-16-01711] McNeish D. (2018). Thanks coefficient alpha, we’ll take it from here. Psychological Methods.

[B15-behavsci-16-01711] McRae K., Gross J. J. (2020). Emotion regulation. Emotion.

[B16-behavsci-16-01711] Moreta-Herrera R., Dominguez-Lara S., Rodas J. A., Sánchez-Guevara S., Montes-De-Oca C., Rojeab-Bravo B., Salinas A. (2022). Examining psychometric properties and measurement invariance of the emotion regulation questionnaire in an Ecuadorian sample. Psychological Thought.

[B17-behavsci-16-01711] Olalde-Mathieu V. E., Licea-Haquet G., Reyes-Aguilar A., Barrios F. A. (2022). Psychometric properties of the Emotion Regulation Questionnaire in a Mexican sample and their correlation with empathy and alexithymia. Cogent Psychology.

[B18-behavsci-16-01711] Preece D. A., Becerra R., Hasking P., McEvoy P. M., Boyes M., Sauer-Zavala S., Chen W., Gross J. J. (2021). The emotion regulation questionnaire: Psychometric properties and relations with affective symptoms in a United States general community sample. Journal of Affective Disorders.

[B19-behavsci-16-01711] Preece D. A., Becerra R., Robinson K., Gross J. J. (2020). The emotion regulation questionnaire: Psychometric properties in general community samples. Journal of Personality Assessment.

[B20-behavsci-16-01711] Putnick D. L., Bornstein M. H. (2016). Measurement invariance conventions and reporting: The state of the art and future directions for psychological research. Developmental Review.

[B21-behavsci-16-01711] Rhemtulla M., Brosseau-Liard P. É., Savalei V. (2012). When can categorical variables be treated as continuous? A comparison of robust continuous and categorical SEM estimation methods under suboptimal conditions. Psychological Methods.

[B22-behavsci-16-01711] Rice S. M., Treeby M. S., Gersh E., Ogrodniczuk J. S., Kealy D. (2018). The emotion regulation questionnaire: ERQ-9 factor structure and measurement invariance in Australian and Canadian community samples. Testing, Psychometrics, Methodology in Applied Psychology.

[B23-behavsci-16-01711] Spaapen D. L., Waters F., Brummer L., Stopa L., Bucks R. S. (2014). The emotion regulation questionnaire: Validation of the ERQ-9 in two community samples. Psychological Assessment.

[B24-behavsci-16-01711] Srivastava S., Tamir M., McGonigal K. M., John O. P., Gross J. J. (2009). The social costs of emotional suppression: A prospective study of the transition to college. Journal of Personality and Social Psychology.

[B25-behavsci-16-01711] Valencia P. D., Canchari E. G., Morin-Huapaya J. C., Blancas-Guillen J., Ccoyllo-Gonzalez L. (2026). Cuestionario simplificado de regulación emocional: Análisis psicométrico inicial en universitarios peruanos. Psicodebate.

[B26-behavsci-16-01711] Van Doren N., Zainal N. H., Newman M. G. (2021). Cross-cultural and gender invariance of emotion regulation in the United States and India. Journal of Affective Disorders.

[B27-behavsci-16-01711] Vizioli N. A. (2024). Argentine adaptation of the emotion regulation questionnaire (ERQ): New psychometric evidence. Current Psychology.

[B28-behavsci-16-01711] Zhao M., Kuan G., Zhou K., Musa R. M., Majeed A. P. P. A., Kueh Y. C. (2024). Psychometric properties and gender invariance of the 8-item emotion regulation questionnaire (ERQ-8) among Chinese university students. PLoS ONE.

