# Supplementary material for: Psychometric Evaluation of the Emotion Regulation Questionnaire (ERQ) in Peruvian Psychology Undergraduates: Factor Structure, Reliability, and Measurement Invariance Across Sex
Source: Behav Sci (Basel). 2026 Sep 21;16(9):1711. doi: 10.3390/bs16091711 (PMC13603680; doi:10.3390/bs16091711)
Supplement: Supplementary file 1 [file behavsci-16-01711-s001.zip › behavsci-4534187-supplementary.pdf]

Table S1. *Participants' district of residence (N = 414).*

District	n (%)
Trujillo	191 (46.1)
Víctor Larco	69 (16.7)
La Esperanza	45 (10.9)
El Porvenir	37 (8.9)
Florencia de Mora	18 (4.3)
Huanchaco	18 (4.3)
Moche	10 (2.4)
Laredo	9 (2.2)
Alto Trujillo	9 (2.2)
Other	8 (1.9)

Note. Percentages may not total 100.0 because of rounding.
